# A Lifestyle Intervention During Pregnancy and Its Effects on Child Weight 2.5 Years Later

**DOI:** 10.1007/s10995-022-03395-5

**Published:** 2022-03-06

**Authors:** Karin Haby, Hanna Gyllensten, Ragnar Hanas, Marie Berg, Åsa Premberg

**Affiliations:** 1 Antenatal Health Care, Primary Health Care, Research and Development Unit, Regionhälsan, Gothenburg, Sweden; 2 Institute of Health and Care Sciences, Sahlgrenska Academy, University of Gothenburg, Gothenburg, Sweden; 3 University of Gothenburg Centre for Person-Centred Care (GPCC), Sahlgrenska Academy, University of Gothenburg, Gothenburg, Sweden; 4 Department of Paediatrics, NU Hospital Group, Uddevalla, Sweden; 5 Institute of Clinical Sciences, Sahlgrenska Academy, University of Gothenburg, Gothenburg, Sweden; 6 Department of Obstetrics and Gynaecology, Sahlgrenska University Hospital, Gothenburg, Sweden; 7 Primary Health Care and Research and Development Unit, Regionhälsan, Gothenburg, Region Västra Götaland Sweden

**Keywords:** Child weight, Diet, Food and nutrition (MeSH), Gestational weight gain (MeSH), Maternal health services (MeSH), Obesity (MeSH), Life style intervention, Pregnancy (MeSH)

## Abstract

**Aim:**

The aim of this study was to evaluate if overweight and obesity in the offspring is reduced by a low-intensity antenatal primary care intervention with focus on diet and physical activity for pregnant women with obesity, comparing children to mothers receiving the intervention with children to mothers who did not.

**Methods:**

This study is a follow-up of children 2.5 years of age after their mothers’ participation in a non-randomised controlled intervention intending to limit gestational weight gain. All study participants received standard antenatal care. The intervention group received lifestyle support via motivational talks with midwife and support from dietician. Data on child weight were collected by medical records, letter and phone.

**Results:**

There was no significant difference between the groups 2.5 years after intervention (International Obesity Task Force ISO-BMI 25 (child BMI corresponding to adult BMI of 25): 20% vs. 21%; ISO-BMI 30: 4.6% vs. 1.3%). The mother’s BMI at the beginning of pregnancy significantly influenced child BMI at 2.5 years (r = 0.13, p = 0.014, r^2^ = 0.017). For each unit of increase in maternal BMI at enrollment, the probability of child ISO-BMI ≥ 25 increased by 7.5% (p = 0.021) and of ≥ 30, by 12.9% (p = 0.017).

**Conclusion:**

The frequency of overweight and obesity of the children at 2.5 years of age was significantly correlated to the mother’s BMI, but not correlated to the mothers’ participation in the antenatal lifestyle intervention. Thus, it seems important to address obesity and lifestyle issues before and between pregnancies.

*Trial registration* The study has been registered at ClinicalTrials.gov, Identifier: NCT03147079.

**Supplementary Information:**

The online version contains supplementary material available at 10.1007/s10995-022-03395-5.

## Significance

*What is already known?* Pregnancy contributes to obesity through postpartum weight retention for the women and for the offspring by heredity. Obesity and excessive gestational weight gain increases health risks for the woman and child. Lifestyle interventions can attenuate gestational weight gain and prevent health drawbacks.

*What this study adds?* This low budget effort, with insights from motivational interviewing and person-centered care and with dietician support, positively affected weight. The women in the intervention managed to keep their gestational weight gain lower, but there was no difference in child BMI 2.5 years after the intervention. However, mother’s BMI at the beginning of pregnancy influenced child BMI at 2.5 years.

## Introduction

Obesity in childhood and adolescence undermines the physical, social, and psychological well-being of children and is a known risk factor for adult obesity and non-communicable diseases (WHO, 2018). The World Health Organization states that there is an urgent need to act to improve the health of coming generations. The proportion of children and adolescents with obesity worldwide has risen from 1% in 1975 to 7% in 2016 (WHO, 2018). In the USA, statistics from year 2018 showed that 13.9% of 2- to 5-year-olds had obesity (CDC, 2018). According to Swedish regional data from 2018, in 4-year olds the rate of obesity was 2%, and of overweight 9% (Götaland, 2018). National data showed that among youth 11 to 17 years of age, the prevalence of obesity was 4%, and of overweight 21%, which meant that 25% of the young population in Sweden had BMI ≥ 25 (Livsmedelsverket, 2018).

Predisposition to obesity may originate very early in the child’s development. In line with the concept of the developmental origins of health and disease, the first 1000 days (i.e., in-utero foetal growth and early infancy) have been described as highly critical in the development of childhood obesity (Barker et al., 2008).

Accordingly, obesity in pregnancy is rising and has become a global public health issue (Graviditetssregistret, 2018). Of women assigned to antenatal care in Sweden in 2018, 27% had overweight (BMI ≥ 25) and 15.4% had obesity (BMI ≥ 30) (2018). The prevalence was higher in pregnant women with lower education and women born outside Sweden (2018). Maternal obesity puts the well-being of the next generation at risk, and is one of the most significant factors leading to negative health consequences for both the pregnancy and the foetus (Marchi et al., 2015). Obesity in the pregnant mother can result in obesity in the offspring (Yu et al., 2013), with further negative metabolic health consequences (Drake & Reynolds, 2010; Yu et al., 2013). Paternal BMI also has an impact and together with maternal BMI it made parental BMI the strongest risk factor for obesity at age 2 to 9 in a European study (Bammann et al., 2014). Likewise, obesogenic societal and home environments are associated with high BMI in the child (Schrempft et al., 2018).

In addition to high maternal BMI, excessive gestational weight gain (GWG) can further aggravate the outcomes for the child. High GWG has been associated with high foetal birthweight (Leonard et al., 2017) and with offspring becoming overweight or obese in childhood and adolescence (Mamun et al., 2014), as well as with cardiometabolic risk factors (Tam et al., 2018).

Lifestyle interventions during pregnancy have been successful in limiting GWG (International Weight Management in Pregnancy (i-WIP) Collaborative Group, 2017; Peaceman et al., 2018) and have been assumed to have positive long-term health effects on the offspring. However, follow-up studies of effective GWG interventions have shown conflicting results in children aged 1- and 2.8 years (Mustila et al., 2013; Tanvig et al., 2014). Thus, it remains to be seen whether prenatal lifestyle interventions can have a favourable effect on the weight of the offspring. Therefore, the aim of this non-randomised controlled study was to evaluate whether an effective low-intensity antenatal primary care lifestyle intervention, focused on healthy diet and physical activity, for pregnant women with obesity could result in a lower proportion of overweight and obesity in the children after 2.5 years compared to standard antenatal care.

## Patients and Methods

**Fig. 1:**
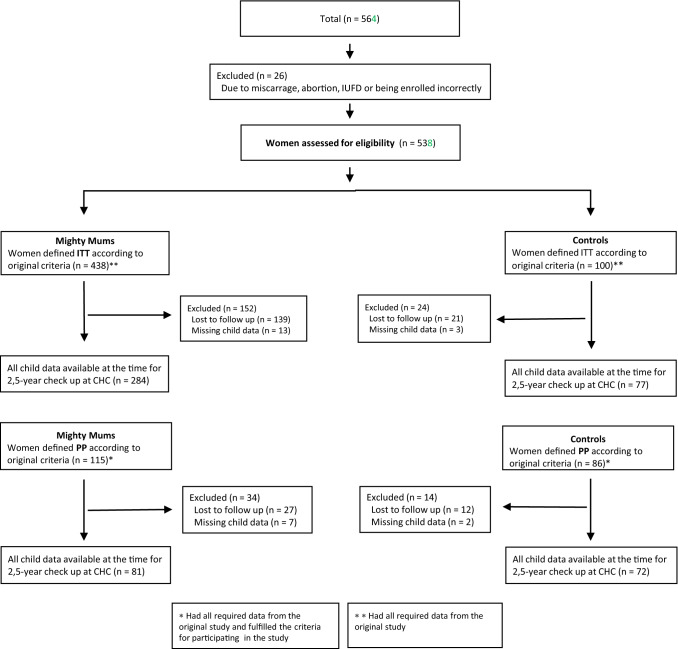
Flow chart of women and children in the study. *ITT* intention-to-treat population, *PP* per-protocol population

The study included children of mothers who participated in an antenatal intervention called Mighty Mums, and who had data available at the follow-up 2.5 years after delivery (Fig. 1). The Mighty Mums project was a non-randomised controlled standardised intervention aimed at reducing GWG in women with obesity delivered during regular antenatal care (Haby et al., 2018). The intervention was conducted in an urban area in western Sweden over 3 years (2011–2013) and was directed towards women entering pregnancy with BMI ≥ 30. After informed consent, women enrolled in the intervention group (n = 438) and in the control group (n = 100) were followed in medical registries and during antenatal care from the first trimester of the pregnancy until the postpartum check-up. The women in the control group were from an adjacent geographical area with a similar sociodemographic structure from a public health perspective.. The second registry control group in the original Mighty Mums project (Haby et al., 2018) did not contribute with child data and was not included in this study on the children, thus reducing the number of controls.

All participants in the project (n = 538) received standard antenatal care. The women in the intervention group (n = 438) were encouraged to gain less than 7 kg during pregnancy. They received additional support, focusing on physical activity and nutritious food, to develop a healthier lifestyle. Information on lifestyle changes was delivered by the midwife at two extra 30 min appointments during early pregnancy. Thereafter, about 5 min of each appointment with the midwife during pregnancy were dedicated to lifestyle follow-up.The women were also offered individual and group sessions with a dietician, prescriptions for physical activity, walking poles, pedometers, and information about community health centres. Adherence to the programme was monitored until postpartum check-up in a log book designed for the Mighty Mums project. The woman’s weight was checked at every appointment including the postpartum check-up approximately 6 weeks after birth.

To address lack of adherence, women were divided into two subpopulations, Intention to treat (ITT) and PP (Haby et al., 2018). All participating women were included as ITT. Both intervention and control group were assigned to PP if they had registered weight and height at their first visit to antenatal care and weight at their last visit. The women in the intervention were also required to participate in the intervention at a defined minimum level (Haby et al., 2018), meaning they had at least partly adhered to defined food and physical activity recommendations on logged antenatal visits during pregnancy. Maximum attendance implied seven notifications in the log book, corresponding to seven discussions on the topic throughout pregnancy until postpartum check-up. In this follow-up study, all children with data for the 2.5 year check-up are included. The same grouping of ITT and PP as in the previous study was used for the children evaluated in the follow-up study, since no additional intervention activity was conducted after childbirth (Haby et al., 2018).

Of the women in the original intervention (ITT, n = 438), 26.5% fulfilled the criterion of adherence to the study protocol, i.e. performed the prescribed activities at level two of four on at least three of six follow-ups with the midwife during pregnancy. All extra activities were optional; 38.8% (n = 170) had contact with the dietician (individually or in food discussion group), 34.7% (n = 152) used pedometers, 20.1% (n = 88) used walking poles and 16.9% (n = 74) participated in aqua aerobics. Most women chose to organise physical activities on their own and the most common activity was walking, often on a level of 30 min 5–7 days a week. The mean number of visits with logbook activity were higher (6.3 ± 0.6) in the PP-population than in the ITT-population (4.7 ± 2.3). Dietician counselling, using walking poles and pedometers as well as participating in aqua aerobics were more common in the PP-population, and they also had a slightly higher score concerning the composite variable for all activities (4.3 ± 1.1 vs. 3.5 ± 1.7). Finally, the women in the PP intervention group were more likely to achieve the recommended < 7 kg GWG (37 vs. 23%), and had significantly lower (2.3 kg) mean GWG (8.9 vs. 11.2 kg) than the control group (Haby et al., 2018).

## Data Collection

To collect the children’s weight and height at 2.5 years, the mothers were contacted at their homes by traditional mail, and if there was no response they were followed up by telephone. All children in Sweden are monitored at set time points according to Swedish standards for child health care. This preventive physical, cognitive, and linguistic surveillance is free of charge and reaches almost 100% of the children. Data is entered in child health care-records and also registered in the child’s health book of anthropometric and other health variables, which belongs to the parents. Birth weight is reported in grams to four valid digits (Table 2). When the child is weighed at 2.5 years, wearing light underwear and standing on a digital scale, its weight is reported in kilograms to one decimal (Table 3). Height is measured in a standardised way, with the child standing up, and is reported in centimetres to one decimal (Table 3). When it was not possible to get the information from the mother via the child’s health book, data on weight and height were collected from the medical records in child health care. The time point 2.5 years of age was deemed appropriate since at this age the child’s food intake is still mainly under control of the parents, and there is no more check-up in child health care until the child is 4 years old. Weight and height for children with visits > 1 month off 2.5 years were extrapolated to the age of 2.5 years according to Swedish growth charts. BMI was calculated as weight in kilograms divided by height in meters squared and expressed as the continuous variable BMI standard deviation score (BMI-SDS), based on age- and sex-specific Swedish growth charts. To categorise obesity, international standards were used to define which child BMI-SDS represented adult BMI cut off levels of 25 and 30 respectively (International Obesity Task Force [IOTF]-BMI 25 and IOTF-BMI 30, also called ISO-BMI 25 and ISO-BMI 30) (Cole et al., 2000) (Table 3).

Baseline data on the women were collected from the antenatal medical records and the National Pregnancy Register. The weight measured at the first antenatal visit was used to calculate baseline BMI. The definition of GWG was the weight difference between the first pregnancy visit and the last pregnancy visit with weight reported to the Register (Table 1). Data for the child on birth weight and breastfeeding at 4 weeks were collected from the clinical record (Table 2).

**Table 1:** Clinical and sociodemographic baseline characteristics of the study participants at the first pregnancy visit, for women contributing with child data at the 2.5 year follow-up

Variable	Intention to treat (ITT) population			Per protocol (PP) population		
	Intervention n = 284	Control n = 77	P	Intervention n = 81	Control n = 72	P
Age, years Mean ± SD (range)	31 ± 5.3 (18.2–47.1)	31 ± 4.8 (21.7–43.3)	0.78	30 ± 5.1 (20.7–42.1)	31 ± 4.9 (21.7–43.3)	0.62
BMI^a^ at enrolment Mean ± SD (range)	34.1 ± 4.0 (28.3–57.2)	34.5 ± 3.7 (29.7–43.9)	0.42	33.7 ± 3.4 (29.3–47.0)	34.2 ± 3.7 (29.7–43.9)	0.35
GWG^b^, kg Mean ± SD (range)	11.0 ± 7.0 (− 24.0–53.0)	11.5 ± 6.5 (0.0–25.0)	0.54	10.4 ± 6.0 (− 4.0–29.0)	11.7 ± 6.6 (0.0–25.0)	0.18
Primipara n (%)	145 (51)	38 (49)	0.80	49 (51)	33 (46)	0.076
Born outside Sweden n (%)	71 (25)	4 (5.2)	< 0.001	17 (21)	3 (4.2)	0.003
Use of interpreter n (%)	17 (6.0)	0 (0.0)	0.029	2 (2.5)	0 (0.0)	0.50
Education^c^, n (%)	162 (57) n = 2 83	49 (64)	0.36	43 (53) n = 76	45 (63) n = 70	0.26
Other than employed^d^ n (%)	82 (29) n = 274	2 (2.6) n = 74	< 0.001	20 (25)	2 (2.8)	< 0.001
Use of nicotine^e^ n (%)	52 (19) n = 280	11 (14) n = 76	0.50	10 (14)	12 (15) n = 71	1.00

In the ITT analysis all individuals with complete data were included, while the PP population excluded women not attending at least half of the planned and logged intervention contacts

^a^BMI (body mass index) was transformed to week 15. Due to clinical routines, BMI was rounded up and some women with a true BMI less than 30 were included (n = 19)

^b^Gestational weight gain was defined as the weight difference between the first and the last pregnancy visit with weight measurement

^c^Education up to university studies

^d^Being subsidised by parental leave, unemployment benefits, student loans, or social security

^e^Cigarettes or snuff

**Table 2:** Clinical baseline characteristics of the children contributing with data at the 2.5-year follow-up

Variable	Intention to treat (ITT) population			Per protocol (PP) population		
	Intervention n = 284	Control n = 77	P	Intervention n = 81	Control n = 72	P
Birth weight Mean ± SD (range)	3590 ± 568 (830–5430)	3690 ± 566 (1840–5130)	0.15	3580 ± 539 (2525–5430)	3740 ± 521 (2330–5130)	0.067
Week of birth Mean ± SD (range)	40 ± 2 (24–42)	40 ± 2 (29–42)	0.67	40 ± 1 (36–42)	40 ± 1 (37–42)	0.533
Macrosomia^a^ n (%)	15 (5) n = 282	6 (8)	0.41	4 (5)	6 (8)	0.52
SGA^b^ n (%)	3 (1) n = 282	0 (0)	1.00	1 (1)	0 (0)	1.00
LGA^b^ n (%)	31 (11)	7 (9)	0.83	7 (9)	7 (10)	1.00
Sex, girls n (%)	147 (52)	40 (52)	1.00	45 (56)	38 (53)	0.75
Breastfed^d^ at 4 weeks, n (%)	206 (73) n = 248	52 (67) n = 63	1.00	65 (80) n = 75	50 (70) n = 60	0.63

In the ITT analysis, all individuals with complete data were included, while the PP population excluded women

not attending at least half of the planned and logged intervention contacts and their children

^a^Birth weight > 4500 g

^b^Small for gestational age

^c^Large for gestational age

^d^Completely or partly

All analyses were performed using IBM SPSS Statistics software v. 25. Statistical analyses were conducted between groups, based on group assignment of the mothers in the original study (Haby et al., 2018). Continuous variables were compared with t-test between the intervention and control groups. Chi-square and Fisher’s exact tests were used for comparisons of categorical characteristics. Pearson’s correlation was used for bivariate comparisons of BMI. ITT and PP were analysed separately. An analysis of covariance (ANCOVA) was used to control for confounding factors identified by comparing baseline characteristics between the intervention and control group (i.e., mother’s BMI, country of birth, use of interpreter, employment status, and use of nicotine). Factors that differed significantly (born outside Sweden, use of interpreter, employment status) were included as adjusting factors in ANCOVA. Since there was no significant difference in the primary outcome, a post-hoc correlation model was used to explore the association between the mother’s BMI at the beginning of pregnancy and the child’s BMI at 2.5 years. Binary logistic regression was used to estimate odds ratios for ISO-BMI.

## Results

The flow chart of the study is shown in Fig. 1. Overall, 538 children were analysed for outcome at birth and 361 (67%) remained for analysis at the age of 2.5 years. Weight and height were collected from the mother for 65% of the children and from medical records for 35%. For 78 of 153 children (age range 28.6 to 32.5 months), weight was extrapolated to the age of 2.5 years. The number of women lost to follow-up was unevenly distributed between the intervention and control groups (35% vs. 30% in ITT and 24% vs. 16% in PP). In the intervention group, women lost to follow-up were significantly more often not born in Sweden, had lower education, used interpreters more, and were less employed. This was not the case in the control group (Supplemental Table 1)*.*

In the PP population, participants from the intervention and control group differed significantly at baseline for country of birth and employment status, and in the ITT population they also differed by use of interpreter (Table 1). There were no significant baseline between-group differences in children’s weight and weeks at birth, macrosomia, small and large for gestational age, gender, or breastfeeding (Table 2).

There were no significant between-group differences for the children’s mean BMI and ISO-BMI at 2.5 years, in either ITT or PP population (Table 3). No significant differences in the proportion of overweight or obesity at 2.5 years were seen (Table 3). The GWG of the mother had no significant effect on the child’s BMI at 2.5 years (ITT: r = 0.036, p = 0.49; PP: r = − 0.03, p = 0.71) and GWG < 7 kg did not have an influence on offspring BMI (ITT: p = 0.32, PP: p = 0.56).

**Table 3:** Results for children at 2.5-year check-up after the intervention

Variable		Intention to treat-population (ITT)			Per protocol-population (PP)			
	Intervention n = 284	Control n = 77	Difference (95% CI)	P	Intervention n = 81	Control n = 72	Difference (95% CI)	P
Weight, kg Mean ± SD (range)	14.5 ± 1.9 (9.8–21.1)	14.8 ± 1.7 (12.1–19.8)	0.17 (− 0.141; 0.791) 0.344 (− 0.138; 0.826)	0.171 (unadj) 0.16 (adj^c^)	14.2 ± 1.8 (9.8–20.1)	14.7 ± 1.7 (12.1–19.8)	− 0.55 (− 1.114; 0.021) 0.35 (− 0.132; 0.840)	0.059 (unadj) 0.15 (adj^c^)
Height, cm Mean ± SD (range)	92.7 ± 4.1 (72.5–102.0)	93.6 ± 3.6 (86.5–100)	0.067 (− 0.066;1.966) 0.801 (− .251;1.853)	0.067 (unadj) 0.14 (adj^c^)	92.0 ± 4.3 (79.0–89.5)	93.7 ± 3.6 (86.5–100.0)	− 1.64 (− 2.916; − 0.370) 0.80 (− 0.260; 1.861)	0.012 (unadj) 0.14 (adj^c^)
BMI Mean ± SD (range)	16.8 ± 1.6 (12.2–23.4)	16.9 ± 1.4 (13.0–20.4)	0.87 (0.369; 0.438) 0.107 (− 0.308; 0.522)	0.870 (unadj) 0.611 (adj^c^)	16.7 ± 1.6 (12.2–22)	16.8 ± 1.4 (13.0–20.4)	− 0.046 (− 0.528; 0.436) 0.12 (− 0.298; 0.539)	0.85 (unadj) 0.57 (adj^c^)
ISO BMI 25^a^ n (%)	57 (20)	16 (21)		0.87	15 (19)	14 (19)		1.00
ISO BMI 30^b^ n (%)	13 (4.6)	1 (1.3)		0.32	2 (2.5)	1 (1.4)		1.00

In the ITT analysis all individuals with complete data were included, while the PP population excluded women not attending at least half of the planned and logged intervention contacts, and their children

No statistically significant differences were identified

^a^The child’s BMI predicted to be ≥ 25 as an adult according to longitudinal growth charts

^b^The child’s BMI predicted to be ≥ 30 as an adult, according to longitudinal growth charts

^c^Adjusted for employment status, use of interpreter, mother being born abroad

Analysis of covariance did not show a statistically significant effect by intervention group (Table 3). However, post-hoc analyses found that the mother’s BMI in the beginning of pregnancy correlated significantly with the child’s BMI at 2.5 years (r = 0.13, p = 0.014, r^2^ = 0.017). Thus, the mother’s BMI at the beginning of pregnancy explained 1.7% of the variation in child BMI at 2.5 years. The probability for a child BMI > 25 increased 7.5% for each BMI unit of mother at beginning of pregnancy (p = 0.021). The probability for child BMI > 30 increased 12.9% for each BMI unit of mother at beginning of pregnancy (p = 0.017).

## Discussion

This study found no decrease in overweight or obesity among the children at 2.5 years after their mothers participated in a lifestyle intervention to limit GWG. Mean BMI and ISO-BMI did not differ between study groups at 2.5 years, and GWG as such or the occurrence of weight gain below 7 kg did not affect the child’s weight outcome. However, maternal BMI at entry of the pregnancy affected the child’s weight at 2.5 years significantly.

A strength of the current child study is that the Mighty Mums intervention was population based, performed in a routine clinical setting and demanded a minimum of extra resources. The women who were eligible to participate were from geographically as well as socio-economically representative areas, and languages other than Swedish were not an obstacle since using interpreters is a regular practice in Swedish antenatal care. Delivering the intervention through the standard antenatal care was expected to avoid biased results caused by an over-representation of highly motivated women.

A weakness of the original project was that it was not randomised. Also, 35% of eligible pregnant women were not invited due to unclear reasons, but not all midvives feel relaxed when addressing women about weight and lifestyle issues (Christenson et al., 2018; Wennberg, 2015). Of the women invited, 62% chose to participate. At the time of follow-up it was possible to contact 65% of the intervention subjects and 76% of the controls from the initial study, which are figures often seen in similar follow-up studies where 35% to 88% success rates have been reported (Dalrymple et al., 2018; Haby et al., 2018). The number of women lost to follow-up was unevenly distributed between groups, with a higher proportion of lost to follow-up in the intervention group. This could be due to women in the intervention group living in more demanding sociodemographic conditions, for example being immigrants with less fluency in Swedish. Families in the intervention group seemed to have changed addresses or moved abroad more often, and to have followed the child health care programme with regular visits less regularly. There was also an uneven distribution of women between the groups at inclusion, since the midwives were less active in recruiting in the control area (Haby et al., 2015). A larger proportion of women recruited in the control group spoke Swedish, which due to communication challenges could have contributed to the absence of between-group differences in results (Wennberg, 2015), however this was controlled for. Moreover, the women in the intervention were less likely to be primipara and had up to seven children compared with the women in the control group, who had at most three children (Table 1). Since less than one third of the women in the intervention group fulfilled the criterion of sufficient adherence to the study protocol, the conclusions of the PP population are drawn from a rather small proportion of those eligible for participation. The large number of women not included, drop outs and lost to follow up could have impacted on the results, hiding an effect of the intervention on child weight.

With an intervention focusing on healthy diet and physical activity it was expected that mothers who changed behaviour and successfully restricted GWG would beneficially influence the weight development of their children at 2.5 years of age. The absence of significant differences between groups may be due to the fact that the intervention was not intense enough, or that the effect of lifestyle changes may not be evident until later in the child’s life. Interventions during pregnancy may be performed too late to be able to positively influence the weight and the health of the child. Already in 2009, a consensus statement from multidisciplinary experts concluded that early life adversities may extend into adulthood and that actions to control pre-pregnancy weight and lifestyle should be emphasised (Poston et al., 2015). Women in the Mighty Mums intervention group expressed in a qualitative study that they wished that the health care providers had also provided support after childbirth to increase the possibility of maintaining a healthy lifestyle in the long run (Dencker et al., 2016). Child health care offers support to the family regarding lifestyle issues, but the women expressed a wish for personal advice for themselves (25).

Our findings are in line with several other interventions performed during recent years that have been unable to demonstrate a difference in child BMI following lifestyle interventions during pregnancy (Mustila et al., 2013; Parat et al., 2018; Patel et al., 2017; Tanvig et al., 2014, 2015) A systematic review has shown that it was not possible to draw any overall conclusions on the influence of antenatal interventions on measures of obesity in early childhood, due to the heterogeneity of the methodology and reported offspring outcomes (Dalrymple et al., 2018). Mustila et al. found no effect on GWG and no difference in child weight, either at birth or at one year. In their study, the intervention consisted of individual counselling on diet and physical activity and group sessions with a physiotherapist and dietician, which is similar to the Mighty Mums project (Mustila et al., 2013). Tanvig et al. performed the ambitious LIPO (Lifestyle in Pregnancy and Offspring) study, with individual dietician support and free membership to a fitness centre, and were likewise unable to detect any effect of the intervention on child weight at the 2.8 year follow-up, despite a difference in GWG (Tanvig et al., 2015). Nor were there any effects on child anthropometrics or body composition (Tanvig et al., 2014). Similar results, with reduced GWG but no effect on offspring, have been found by others (Mustila et al., 2013; Tanvig et al., 2014, 2015). However, Patel et al. managed to show significantly lower subscapular skinfold thickness among the children of mothers in the intervention at 12 months (Patel et al., 2017), and Vesco et al. showed a significantly lower weight-for-age z-score at 12 months (Vesco et al., 2016). High (67–80%) adherence to the antenatal intervention protocol has been suggested as a reason for success in limiting GWG (Nagpal et al., 2017). The PP in the Mighty Mums study had a predefined inclusion level of “partly following” the protocol, and it is possible that a higher predefined prerequisite could have resulted in a larger difference in GWG as well as outcome differences at 2.5 years, although that would instead have resulted in a highly selected study population that would not be representative of all pregnant women with obesity. Since only a third of the women managing to reach the level of participation predefined for PP, this might implicate that the programme was not appealing or that the timing was not right due to challenges with being pregnant.

It has been suggested that limiting prenatal weight would be more successful than reducing GWG, but such interventions are difficult to conduct since not all pregnancies are planned (Forsum et al., 2013), and lifestyle interventions after and between pregnancies might present a more effective way to support women to reduce weight successfully (Bertz et al., 2015). Suggestions for potential ways to handle the increasing rate of childhood obesity has been to include more professionals in health care and different parts of the community (Dalrymple et al., 2018). It has also been suggested that it is necessary to reach a larger target population, including the whole family, and to carry out the interventions multiprofessionally throughout the whole health care chain (Campbell et al., 2016). The becoming father, the spouse or a relative often joins the woman on her visits to antenatal care and was welcome to participate also on the extra visits concerning the project, but only a few used this possibility. Maybe a more active involvement with the partner and the rest of the family could have facilitated lifestyle changes for the woman.

## Conclusions

This study indicates that the mother’s BMI at the beginning of pregnancy is associated with the child’s BMI, but the lifestyle intervention performed for pregnant women with obesity could not demonstrate an effect on offspring’s BMI at 2.5 years of age, regardless of a reduced GWG in the intervention group. This finding contributes to a growing body of evidence that interventions during pregnancy to prevent childhood obesity are performed too late, and that reducing the prevalence of pre-conceptional or postpartum obesity may be more effective. However, the fact that the intervention group had more challenging sociodemographic statuses could have offset the intervention effect. Further studies are needed regarding the effect of prenatal lifestyle interventions on health behaviour and weight before pregnancy, as well as on GWG. Also, how a larger proportion of women can be attracted and fulfill lifestyle interventions like this needs to be investigated.

## Supplementary Information

Below is the link to the electronic supplementary material.

Supplementary file1 (DOCX 13 kb)

## Data Availability

The study protocol, statistical analysis plan and informed consent form (all in Swedish) can be requested from the corresponding author. The individual data in this study are not publicly available. Data can only be available after legal review to researchers who meet the criteria for access to this type of sensitive and confidential data (in accordance with the Swedish Ethical Review Act, the Personal Data Act, and the Administrative Procedure Act).
