# Supplementary material for: A Lifestyle Intervention During Pregnancy and Its Effects on Child Weight 2.5 Years Later
Source: Matern Child Health J. 2022 Mar 6;26(9):1881–90. doi: 10.1007/s10995-022-03395-5 (PMC9374787; doi:10.1007/s10995-022-03395-5)
Supplement: Supplementary file 1 — Supplementary file1 (DOCX 13 kb) [file 10995_2022_3395_MOESM1_ESM.docx]

Supplemental Table 1. Significance of lost to follow-up

|  | **Intention to treat(ITT) population** | | | **Per protocol (PP) population** | | |
| --- | --- | --- | --- | --- | --- | --- |
| Variable | **Intervention**  (N = 284) | **Control**  (N = 139) | **Intervention**  (N = 81) | | **Control**  (N = 27) | |
| Born outside Sweden  Use of interpreter | 0.008  0.002 | n.s.  n.s. | 0.003  0.001 | | n.s.  n.s. | |
| Education below university level | 0.011 | n.s | 0.046 | | | n.s. |
| Being subsidised by parental leave, unemployment benefits, student loans, or social security | 0.006 | n.s. | n.s | | | n.s. |
